# A Tribute to the Global Health Legacy of Jimmy and Rosalynn Carter

**DOI:** 10.4269/ajtmh.23-0641

**Published:** 2023-10-04

**Authors:** Kashef Ijaz, Julie Jacobson

**Affiliations:** ^1^ The Carter Center, Atlanta, Georgia;; ^2^ Bridges to Development, Vashon, Washington;; ^3^ American Society of Tropical Medicine and Hygiene (ASTMH), Arlington, Virginia

**Figure:**
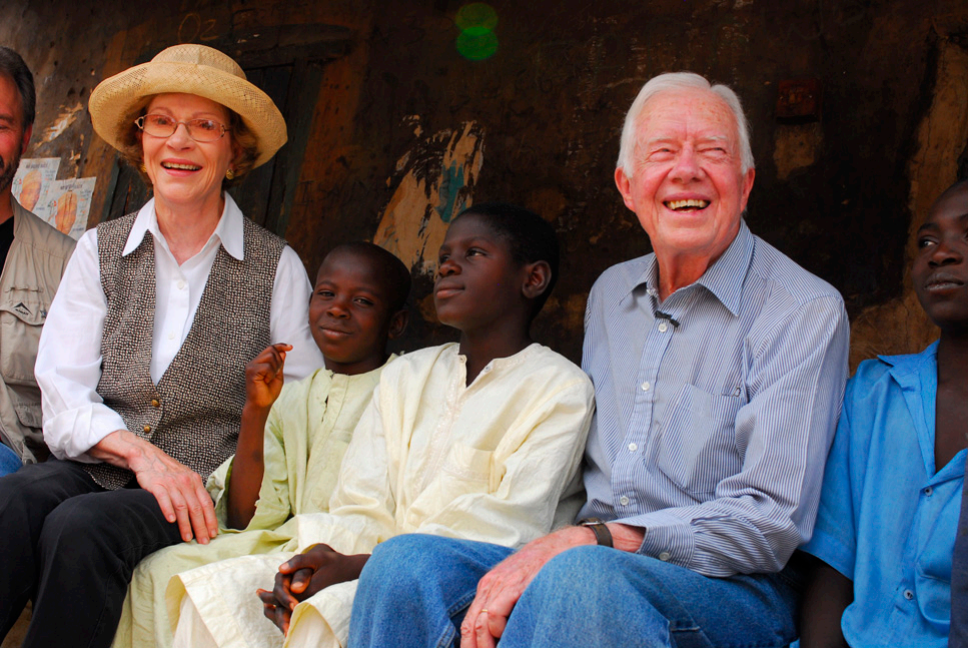
For decades, former U.S. President Jimmy Carter and First Lady Rosalynn Carter traveled to observe first-hand the impact of the Carter Center-assisted health programs and bring attention to countries’ needs and community progress. In Nigeria, President and Mrs. Carter met with children who received treatments and health education to prevent neglected tropical diseases. Credit: The Carter Center — E. Staub.

The global public health legacy of former U.S. President Jimmy Carter and First Lady Rosalynn Carter cannot be overstated. For more than 50 years, they have leveraged the power of their characters and connections to advance public health in the United States and around the world.

President Carter’s foreign policy emphasized human rights, and the Carters have always maintained that health is a human right. President Carter shifted the U.S. Agency for International Development away from technical and capital assistance programs, focusing instead on basic human needs such as food and nutrition, population planning, health, and education.

After his presidency ended, these priorities became hallmarks of The Carter Center, manifested in numerous programs promoting peace through democracy, human rights, rule of law, and conflict resolution; eliminating and eradicating tropical diseases; and advancing mental health reform.

Through The Carter Center and beyond, President and Mrs. Carter have championed progress across many of the focus areas of the American Society of Tropical Medicine and Hygiene (ASTMH). They have used the ASTMH platform and the talent and reach of its members to extend and share their work through the ASTMH Annual Meeting and the *American Journal of Tropical Medicine and Hygiene*. President Carter spoke at the ASTMH Annual Meeting in 2001, sharing his views and experience, and inspiring others to carry on.

The Global Guinea Worm Eradication Program has been the Carter Center's flagship health program. In 1986, when the Center took up this work, an estimated 3.5 million people per year were infected with the loathsome meter-long worm. In 2022, only 13 human cases were recorded on a planet of 8 billion people.^1^ Tens of millions of cases have been averted since this work began.

This success was not accomplished with any vaccine or drug, but through community engagement and basic public health interventions to identify cases, manage these cases, and treat water sources to interrupt parasite transmission in some of the most difficult settings around the world. Hundreds of thousands of volunteers in tens of thousands of remote villages received training in how to filter water to make it safe to drink and how to manage infected people and animals to prevent transmission. These lessons transcended borders, distances, generations, language, and cultural differences, bringing the world to the brink of eradicating this torturous worm.

In 1995, as part of the Carter Center campaign, President Carter used his remarkable diplomatic ability to negotiate a humanitarian cease-fire in the long Sudan Civil War to allow health workers to enter the combat zone to fight Guinea worm. It became known as the Guinea Worm Cease-fire. At the time, it was the longest humanitarian truce in world history. The cease-fire also allowed health teams to administer polio vaccinations and ivermectin (donated by Merck) for river blindness.

The Carters often traveled together to oversee the work of, and drum up support for, various Carter Center health programs around the world. And when they traveled, they listened. When WHO Director-General Tedros Adhanom Ghebreyesus was Ethiopia’s Minister of Health, he told President Carter about difficulties in controlling malaria in the country. Even though the Center wasn’t working on malaria in Ethiopia, it joined with other donors to provide the country with sufficient insecticidal bed nets, leading to a remarkable reduction in the disease.

President Carter likes to say, “There are no neglected diseases, only neglected people.” Rather than neglect the people “at the end of the road,” President and Mrs. Carter and The Carter Center have embraced, partnered with, and empowered them. As President Carter said in this Journal in 2022, “Regardless of differences, people will improve their own lives when provided with the necessary skills, knowledge, and access to resources.”^2^

Mrs. Carter, who has worked tirelessly on mental health issues since even before her husband was president, applied that same principle in Liberia. The Carter Center’s Mental Health Program helped Liberia build a mental health care system from the ground up, including working with the government to build a curriculum to train hundreds of nurses and midwives as mental health clinicians.

Whichever the country, it is community-based staff and volunteers who make the Center’s health programs work. They are the ones delivering health education, engaging in disease surveillance, and distributing medications to their neighbors, friends, and families.

With its deeply invested partners, The Carter Center has assisted national health ministries and communities in achieving a list of impressive accomplishments:

- Scaling up and then scaling down mass drug administration programs for river blindness, trachoma, and lymphatic filariasis, leading to the elimination of:
  ○ River blindness transmission in Ecuador, Colombia, Mexico, and Guatemala, in parts of Nigeria and Ethiopia, and in almost all of Uganda;
  ○ Lymphatic filariasis as a public health problem in large parts of Nigeria, Ethiopia, and Hispaniola; and
  ○ Trachoma as a public health problem in Ghana and Mali.
- Hundreds of thousands of surgeries performed to reverse the effects of advanced trachoma.
- Near elimination of malaria in the Dominican Republic.
- Improvements in health career training in Nigeria, Ethiopia, and Sudan.
- Health systems strengthening everywhere the Center assists.

All of this was made possible by the unwavering vision and seemingly inexhaustible energy of President and Mrs. Carter. Beyond Carter Center programs, the Carters have been champions for the global health agenda, using their enormous prestige and access to engage national and international leaders, corporate directors, donors, scientists, and politicians to bring their own unique qualities and experiences to bear on immense challenges.

President and Mrs. Carter now can watch the impact of their work be magnified through the many people they have touched over their lives, including many of the ASTMH family. At the ASTMH Annual Meeting in Chicago in October 2023, their work, lives, and linkages to the ASTMH mission will be shared in the closing plenary session where we hope to build on their momentum to inspire action and accelerate progress into the future.
